# Supplementary material for: Stimulus selection enhances value-modulated somatosensory processing in the superior colliculus
Source: PLoS Biol. 2025 Mar 31;23(3):e3003057. doi: 10.1371/journal.pbio.3003057 (PMC12135940; doi:10.1371/journal.pbio.3003057)
Supplement: Attachment — Submitted filename: ReviewerResponse_auresp_3.docx [file pbio.3003057.s010.docx]

Reviewer #1 (Michael Brecht): In their paper 'Stimulus selection drives value-modulated somatosensory processing in superior colliculus'Chu et al. record responses from tactile neurons in the mouse somatosensory cortex and superior colliculus during to go (lick reward) or nogo (nonreward) stimuli. The authors find largely symmetric go / nogo tactile responses in somatosensory cortex, but see a heavy bias to the reward predicting go stimulus in the colliculus. I think this is an interesting study. The strengths of the work are the following: (i) The simultaneous recording of two brain structures reveals important insights here. (ii) The somatosensory collicular representations are heavily understudied. (iii) The data are convincing. (iv) The effects are interesting and the reward bias in the colliculus makes biological sense.

Thank you for recognizing the value of our work.

My criticisms are minor:

1. The authors cite broadly and correctly most of the relevant literature but missed two highly relevant references from the primate visual system.

Horwitz, G. D., & Newsome, W. T. (1999). Separate signals for target selection and movement specification in the superior colliculus. Science, 284(5417), 1158-1161.

Horwitz, G. D., & Newsome, W. T. (2001). Target selection for saccadic eye movements: direction-selective visual responses in the superior colliculus. Journal of Neurophysiology, 86(5), 2527-2542.

Thank you for the suggestions, we have added them to the Introduction.

2. Individuals might differ in this task. In general, I think the data are convincing, but I would prefer to see two more S1 animals (i.e., a comparison of 6 and 6 mice rather than of 6 and 4 mice). Just to be sure.

2 more S1 mice have been added as requested for the detection task (Fig 3). S1 maintained symmetric bias with 6 animals. Figure 3 has been updated.

3. Did the authors ever poke into collicular tongue representation? See. Ito, Brendan S., et al. "A collicular map for touch-guided tongue control." bioRxiv (2024): 2024-04. This might provide further inside here.

This is an interesting question. However, it appears that the whisker representation in SC is distinct from the tongue region, as we found very few (<5) neurons in our dataset that had activity related to the onset of licking. I think the whisker region is more medial.

Reviewer #2 (Saba Gharaei): In this study, the authors reveal how the representations of sensory space in S1 and SC are modulated by behavioural context. The authors recorded neuronal activity in S1 and SC of awake behaving mice performing a stimulus selection task that was guided by value-based associations. The authors found that stimulus value and its associated action did not modify the representation of sensory space in S1. However, the behavioural context influenced the representation of sensory space in SC where neuronal responses were biased towards the positive valued stimulus.

When the opportunity to select the positive valued stimulus was removed, this bias in SC neurons was reduced. The experiment has an elegant design whereby the opportunity for mice to select the positive valued stimulus is removed with alternating trials in Home and Away conditions. Overall, this is a timely and well-performed study, the analyses seem appropriate, and the conclusions are supported by the neuronal and behavioural data.

Thank you.

I have only a few minor comments to enhance the clarity of the paper.

For the Hits vs Miss (or removing the opportunity to lick), the authors focused their analysis on neurons that preferred the positive stimulus. This is a logical choice. The authors demonstrate that for these SC neurons, the response to the positive stimulus was often larger on Hit trials compared to that of the Miss or Away trials. It is not clear to me whether the authors also looked at the activity of the same positively preferring neurons, to compare correct rejection trials and false alarms (licking to the no-go stimulus). It would be informative to see if the observed suppression of responses to the "no-go" stimulus is absent on trials that the animal made an error and licked to the no-go stimulus.

Thank you for the suggestion. We have a limited number of false alarm (FA) trials, but we did our best to analyze them. In one mouse, we had a decent number, and we now show those results in Suppl. Fig. 1H, I. Across the population of mice, using the limited number of trials, our results show that firing rates during FA trials were nearly identical (no signif different) to CR trials and were much smaller than Hits.

We have added,

“Consistent with the sensory origin of the observed effects, SC activity was unmodulated by lick initiation, and neuronal firing rates on false alarm trials were significantly smaller than Hits (Suppl. Fig. 1G – I)”

More details of recording sites should be included. What was the depth of recordings? For S1, was the recording across all layers? For SC, was the recording only across the intermediate layers that are sensitive to somatosensory stimuli or across all layers of SC including the superficial and deep? It would be good to include this information given the observation that most SC neurons (76%, 549/727) displayed a significant tactile response, despite the fact that only 2 whiskers contacted the pole.

We recorded primarily from the intermediate and deep layers of the SC. We recorded from all layers of the cortex. This is now described in Suppl. Fig. 1B. Due to the lateral region of our SC penetration, only a small fraction of the electrode sites was in the superficial layer. We only analyzed neurons with a significant tactile response. Potential visual stimuli were blocked by covering the right eye with an opaque object.

By comparing the peak responses in Fig 2B (value-based task) and 3C (equal value task), it is clear that the stimulus bias in SC, was due to the reduction of activity to the "no-go" stimulus rather than enhancement of responses to "Go" stimulus. Perhaps this could be made more explicit. Indeed, it was nice to see that this was further confirmed by the analyses in Fig 4.

Yes, this is true. To further describe this effect, we calculated the firing rate of Positive preferring (during stim selection) and Whisker 1 preferring neurons (during stim detection) relative to their baseline spike rate. This analysis shows that the preferred responses in stim selection and detection mice are similar. However, the non-preferred responses are significantly different between selection and detection mice. In detection mice, sensory suppression is weakly related to baseline spike rate. This result is shown in Supp. Fig. 3F.

We added,

“Touch suppression to non-preferred stimuli and its interaction with baseline spike rate was significantly reduced in animals performing the stimulus detection task (Suppl. Fig. 3F), suggesting that the suppression of high firing rate neurons could be important for ignoring an unwanted stimulus.”

It would be useful to include further details about the behavioural methodology. How many days did it usually take for the training of mice for the whisker discrimination and the whisker detection task?

Thank you for the suggestion. This and other behavioral metrics have been moved to Figure 1.

In the method section, it would be useful to add where on the skull the head-bar was cemented to (i.e. describing Fig1D). As the same animals were used for both SC and S1 recordings (with SC being posterior and BC being anterior), it would be useful for readers to know more details of the custom-built head-bar (e.g. size).

Extra information on the headplate has been added to the Methods.

Reviewer #3: In this manuscript, the authors recorded single-unit activity in the superior colliculus (SC) and the somatosensory cortex (S1) while mice performed a go/no-go whisker discrimination task, allowing them to compare neural activity between the two brain areas during goal-directed behaviours. They found that the firing of SC neurons during whisker stimulation was biased towards the stimulus associated with reward (positive stimulus), while the S1 population had no such bias, consistent with the somatotopic map. Specifically, more SC neurons were positive stimulus-preferring, and their bias was stronger than in S1. To rule out the possibility that this result was driven by a sampling bias, the authors trained mice on a version of the task where both stimuli were associated with reward (detection task). In this task, the SC population showed little stimulus-bias in activity during touch. Further analysis of neural responses in the go/no-go task showed that the SC bias during touch was related to increased spiking on hit trials and delayed suppression of spiking on correct rejection (CR) trials in the positive stimulus-preferring population. Importantly, by comparing hit, miss, and away trials (i.e. where the waterspout was displaced so the animal couldn't respond), the authors found that positive stimulus preference in SC neurons is at least in part due to movement preparation. They also showed that baseline firing rates of SC neurons were lower during miss and away trials than engaged trials (hit + CR), and their baseline firing was correlated with response latency. From this, the authors concluded that SC somatosensory processing is modulated by value during stimulus selection, in contrast with the purely somatotopic mapping of S1.

The authors interpreted the bias in SC responses towards the positive stimulus as sensory encoding modulated by value. However, it is unclear if the bias was indeed related to sensory processing or was purely driven by movement preparation. The authors found that positive stimulus-preference in SC neurons was lower in no movement trials (miss and away), which is still consistent with the movement preparation interpretation. This seems plausible considering that the stimulus bias was quantified over a large time window from touch onset (multiple hundred milliseconds). This time window could include both early sensory and later movement preparation signals. The detection task used in this study did not allow the authors to fully disentangle the contribution of movement preparation to SC responses. Below we suggest some further analyses to address this issue. We recommend that the main conclusions of the paper, including the title and the abstract, be revised based on the results of these analyses. If no further evidence is found to support the SC value mapping interpretation, the conclusions should be adjusted to acknowledge the contribution of movement preparation. If these further analyses indeed support the current conclusions, this paper would provide novel insights into the role of SC in value processing for controlling actions.

1. The authors should analyse whether the performance difference between recording sessions (Fig. S1D) is reflected in the neural responses in SC (i.e. compare hit and CR responses in good and bad performance sessions between mice). If the positive stimulus bias in SC correlates with performance, this would support the value interpretation.

As per your suggestion we have re-calculated the positive stimulus bias for each mouse and found a weak, positive correlation with performance but only for the small d-prime range (<2.5) where there are enough incorrect trials to show a meaningful change in bias. Importantly to note, all stimulus biases shown in the main figures are calculated using correct Hit trials and CR trials where poor performing trials were omitted (we removed CR trials located between consecutive misses, see methods). In the graph below, each point is an individual mouse.

2. Neurons with early activity are likely to have sensory responses as opposed to movement preparation. The authors should quality stimulus bias in a shorter time window from first touch (like Fig. S3E but selecting neurons based on the early period only). Is the positive bias still enhanced in SC when only considering early responses (i.e. sensory neurons)? If yes, the main conclusion would be more supported.

Thank you for the suggestion. We have re-analyzed the stimulus bias of neurons using only the first 50 ms of the sensory response. Positive stimulus bias in the SC was still significantly (p < 0.0001) enhanced over all other stimulus biases. This data is now presented in supplemental Figure 1F and added to the Results. We restricted our analysis to neurons with a significant response during the first 50 ms post first touch.

3. From Fig. 5 the authors concluded that "sensorimotor processing in SC neurons is augmented by movement preparation", but it is possible that they are simply quantifying move preparation per se. They also later state that "positive preferring neurons may represent a specialized population of movement related neurons", which suggest they may agree with the movement preparation interpretation. The authors should also perform the analyses on the Away condition (e.g. Fig. 5E) on early sensory neurons to help clarify this, and they should make the text more consistent.

Thank you for the suggestion. We performed the suggested analysis, looking at only the first 50 ms of the response. During the Away condition, the early response (first 50 ms) is still significantly smaller than during the Home condition. We restricted our analysis to neurons with a significant response during the first 50 ms post first touch.

Action taken in Results,

“Positive stimulus bias in SC neurons significantly decreased on Away trials, while in S1, the effect was significant but very small (Fig. 5H). Despite the decrease during Away trials, the stimulus bias of positive preferring SC neurons was still uniquely large (Fig. 5I). To determine if the early sensory response decreased during Away trials, we restricted our analysis to the first 50 ms post-touch. During this early period, the Hit response and positive stimulus bias significantly decreased (p = 2.8e-6 for Hit response; p = 1.7e-7 for stim bias; n = 237, signed rank test).”

4. The authors should characterise neural dynamics on hit vs false alarm trials, where animals perform the same action to different stimuli. This could also help establish if the SC responses are value-modulated sensory or motor planning signals.

Thank you for the suggestion. We have now analyzed the false alarm (FA) trials, comparing them to CR trials. We found a slight (insignificant) increase in firing rate on FA trials relative to CR trials. Firing rates during FA trials were much smaller than Hit trials. Therefore, the decision to lick on FA trials had only a marginal effect on the sensory response. This data is now presented in Suppl. Fig. 1H, I.

5. All neural data presented are aligned to first touch. Another approach to address whether SC neurons are sensory- or movement-related is to try different alignments of the neural data (i.e. first lick instead of first touch), and compare selectivity strength with different alignments (<https://nam04.safelinks.protection.outlook.com/?url=https%3A%2F%2Fjournals.physiology.org%2Fdoi%2Ffull%2F10.1152%2Fjn.00508.2004&data=05%7C02%7Cspluta%40purdue.edu%7Ca9606a7c43c044d9e8d108dce8f65057%7C4130bd397c53419cb1e58758d6d63f21%7C0%7C0%7C638641391843094694%7CUnknown%7CTWFpbGZsb3d8eyJWIjoiMC4wLjAwMDAiLCJQIjoiV2luMzIiLCJBTiI6Ik1haWwiLCJXVCI6Mn0%3D%7C0%7C%7C%7C&sdata=yxdflD3h53jEhZdzZhDYh0TE9WNZ5vyHwUgOttsOSxw%3D&reserved=0>).

Thank you for the suggestion. We have now calculated a “lick response bias” using the positive preferring SC neurons. We aligned spiking to the onset of first lick during positive stimulus trials and calculated its bias relative to the same period of CR trials. We found that SC spiking was totally unbiased around the onset of the lick response. We spent considerable effort looking at all the single neuron firing rates, to see if ANY neuron could be driving lick onset. We found maybe (if we squint) a few neurons with activity related to lick onset. Therefore, the firing rates of positive preferring SC neurons are unmodulated around the onset of licking. These results are presented in Suppl. Fig. 1G.

Other suggestions:

1. The authors should provide more details on how they ensured that the sampling of neurons that respond to either whisker in the pair is balanced (like they already do for S1). Recordings from naïve animals could address this question.

Thank you for the suggestion. We now show the whisker preference of neurons in SC and S1 as a function of location on the electrode. These data show that whisker preference systematically changes as a function of somatotopic location in mice performing detection (both Go stimuli). These results are now presented in Suppl. Fig. 2.

More importantly, our results are not dependent on our ability to record from equal proportions of neurons that prefer each whisker. We calculate the stimulus bias of each neuron individually, based on its particular whisker preference. Our conclusions would only be problematic if all our recorded neurons preferred the same whisker. This is clearly not the case.

2. The paper would benefit from more plots that show neural data subject-by-subject (e.g. for Fig 2C and E), especially because S1G suggests there is considerable variance in touch rates across mice.

Thank you for the suggestion. We analyzed each mouse separately and found that touch rate bias was unrelated to stimulus bias in SC and S1. These results are now presented in Suppl. Fig. 1.

3. The authors concluded from Fig. 4G-I that "touch suppression in SC may be computed from a different circuit than touch facilitation". That seems like a strong and not necessarily correct interpretation. Their interpretation in the Discussion, which offers both different circuits and local inhibition as possible mechanisms, seems more plausible.

The aforementioned sentence in the Results has been deleted.

4. The trial numbers in Fig. 5 and Fig. 6 seem to be different across contexts (hit/engaged, miss, away). The authors should match trial numbers (i.e. subsample from the hit/engaged and away states) to see if the conclusions still hold.

We resampled the data 100 times for each condition to estimate the mean effect when the trial numbers are the same for the Hit and Away/Miss conditions. The results still hold, as shown in the figure below.

Change in sensory-evoked and spontaneous firing rates during Away and Miss trials after resampling the data to match trial numbers. A) Change in the sensory response. B) Change in spontaneous firing rate. All changes in firing rate are significantly different from zero.

5. Related to Fig. 6, the authors state that "Baseline SC firing rates predict choice probability and speed". To make statements about choice probability, the authors would need to only compare miss and hit, rather than miss and engaged (hit + CR).

We no longer claim SC activity is related to choice probability, and now we discuss the opportunity to respond.

6. The clarity of the data presentation could be improved, especially the following panels:

a) It is confusing how Fig. 4D is different from Fig. 2B. The authors should explicitly state that the neurons in Fig. 4D are sub-selected based on baseline firing >4Hz and should provide a similar plot for the neurons <4Hz.

We have now made it clearer and added the BFR < 4 Hz data to Fig. 4D.

b) Can the authors make it clearer how the population of neurons in Fig. 4G relates to the ones in the other panels of Fig. 4?

They passed the test for significant facilitation/suppression from baseline. This is stated in the figure legend and the Results.

Minor comments:

1. It would be helpful to show task performance (S1B-D) in the main figure.

We have now included this in Fig. 1.

2. The authors could statistically test whether SC has more positive encoding neurons compared to S1 (e.g. Chi squared test), rather than simply showing the percentage of neurons.

We have added this chi-square test to the Results when describing the proportion of neurons. Please note that our conclusions don’t rest on our ability to record from equal proportions of stimulus preferences. We are mainly showing that stimulus bias is uniquely large for SC neurons that prefer the positive whisker.

3. The authors could consider showing significance in the plots and not only in the text to make it easier for the reader to follow (e.g. Fig. 2C, E).

Added. Thanks.

4. Typo in S3 title: "stimulus bias" instead of "stimulus".

Fixed. Thanks.
